# São Paulo Medical Journal: heading towards the 80th anniversary

**DOI:** 10.1590/S1516-31802011000200002

**Published:** 2011-03-03

**Affiliations:** I Associate Professor in the Discipline of Thoracic Surgery, Hospital das Clínicas, Faculdade de Medicina, Universidade de São Paulo (HCFMUSP), São Paulo, Brazil.; II Physician and Preceptor of the Discipline of Thoracic Surgery, Hospital das Clínicas, Faculdade de Medicina, Universidade de São Paulo (HCFMUSP), São Paulo, Brazil.

In 2010, the Associação Paulista de Medicina (APM) reached its 80th anniversary. Over these years, one of its main objectives has always been to improve the technical-scientific knowledge of its associates and provide continuing medical education. Today, we have two scientific journals available, with very distinct purposes: *Diagnóstico e TratamentoandSão Paulo Medical Journal — Evidence in Healthcare.* The São Paulo Medical Journal was founded in 1932 and is one of the oldest publications in Brazil. It is produced every two months and is indexed in several databases, such as Lilacs (Bireme), SciELO and Medline. In 2009, the first impact factor for the São Paulo Medical Journal was published by the Institute of Scientific Information (ISI) - Web of Knowledge and Thomson Reuters: 0.746. This places our journal among the most highly rated journals in Brazil, and in evidence on the worldwide stage.

In 2008, Brazil had 31 journals indexed in ISI, but in 2009, this number went up to 72, i.e. an increase of 132%.^1^ This growth was certainly due to many factors, involving qualification systems, government policies and backing, and persistent authors and editors. We have grown a lot, but how can we grow further?

In 1899, Elbert Hubbard wrote a short story that became famous around the world, with translations into 37 languages. It became a well-known allusion within American popular and business culture until the middle of the twentieth century. In "A Message to Garcia",^2^ after war broke out between the United States and Cuba, the American President needed to communicate rapidly with the leader of the rebels, Garcia. This person was in a fortress in the Cuban backcountry, and no one knew exactly where he was. Someone reminded the President that only Rowan would be able to find Garcia.

Rowan was brought to the President, who entrusted a letter to him, with the task of delivering it to Garcia. Rowan took the letter, put it into a waterproof envelope, tied it to his chest and, after four days, leapt out onto the Cuban coast in the middle of the night to fulfill his role. This he did without harboring any doubts or even asking for any clues regarding Garcia's whereabouts. The tale is short, but it carries a worthy message. Taking the message to Garcia signifies delivering the goods: carrying out the task in an obstinate and objective manner, without excuses for doing the work that has to be done.

We decided to cite A Message to Garcia because we believe that we have reached an important point in the history of our journal. With indexation in Medline and ISI, the São Paulo Medical Journal is certainly made more attractive both for Brazilian and for foreign authors. In fact, we have already received several studies from researchers in Latin America, Europe, Asia and Oceania, and this number is expected to progressively increase. It is up to us at the São Paulo Medical Journal, i.e. editors, members of the editorial board and reviewers, to continue to work towards enabling our journal to continue to grow and become faster and more efficient, such that in the coming years we can be on an equal footing with the best international periodicals. In this endeavor, the role of Brazilian national researchers is of prime importance. Many of the studies presented at our congresses are not even submitted for publication in any periodical. The São Paulo Medical Journal today is an excellent vehicle for publishing our science, and it is only through combined effort that we will be able to achieve our objectives and attain the desired projection.

We invite all researchers, postgraduate students and scientists to submit their papers to the São Paulo Medical Journal. We thank our employees, reviewers and members of the editorial board for their tireless work. We also thank the entire directorate of the APM for their technical, political and financial support, which has made it viable to continually improve our journal. With everyone's support, the São Paulo Medical Journal will increasingly be the fundamental vehicle for Brazilian medicine. We will take the message to Garcia in 2011. Let us go forth with Rowan! We will place the letter in our folders and set out heading for the objective that has been outlined.

A happy 2011 to everyone!
